# Local convergence of tensor methods

**DOI:** 10.1007/s10107-020-01606-x

**Published:** 2021-01-04

**Authors:** Nikita Doikov, Yurii Nesterov

**Affiliations:** 1 Institute of Information and Communication Technologies, Electronics and Applied Mathematics (ICTEAM), Catholic University of Louvain (UCL), Louvain-la-Neuve, Belgium; 2 Center for Operations Research and Econometrics (CORE), Catholic University of Louvain (UCL), 34 voie du Roman Pays, 1348 Louvain-la-Neuve, Belgium

**Keywords:** Convex optimization, High-order methods, Tensor methods, Local convergence, Uniform convexity, Proximal methods, 90C25, 90C06, 65K05

## Abstract

In this paper, we study local convergence of high-order Tensor Methods for solving convex optimization problems with composite objective. We justify local superlinear convergence under the assumption of uniform convexity of the smooth component, having Lipschitz-continuous high-order derivative. The convergence both in function value and in the norm of minimal subgradient is established. Global complexity bounds for the Composite Tensor Method in convex and uniformly convex cases are also discussed. Lastly, we show how local convergence of the methods can be globalized using the inexact proximal iterations.

## Introduction

**Motivation** In Nonlinear Optimization, it seems to be a natural idea to increase the performance of numerical methods by employing high-order oracles. However, the main obstacle to this approach consists in a prohibiting complexity of the corresponding Taylor approximations formed by the high-order multidimensional polynomials, which are difficult to store, handle, and minimize. If we go just one step above the commonly used quadratic approximation, we get a multidimensional polynomial of degree three which is never convex. Consequently, its usefulness for optimization methods is questionable.

However, recently in [18] it was shown that the Taylor polynomials of *convex functions* have a very interesting structure. It appears that their augmentation by a power of Euclidean norm with a reasonably big coefficients gives us a global upper *convex* model of the objective function, which keeps all advantages of the local high-order approximation.

One of the classical and well-known results in Nonlinear Optimization is related to the local quadratic convergence of Newton’s method [13, 19]. Later on, it was generalized to the case of *composite* optimization problems [14], where the objective is represented as a sum of two convex components: smooth, and possibly nonsmooth but simple. Local superlinear convergence of the Incremental Newton method for finite-sum minimization problems was established in [24].

The study of high-order numerical methods for solving nonlinear equations is dated back to the work of Chebyshev in 1838, where the scalar methods of order three and four were proposed [2]. The methods of arbitrary order for solving nonlinear equations were studied in [6].

A big step in the second-order optimization theory was made since [22], where Cubic regularization of the Newton method with its global complexity estimates was proposed. Additionally, the local superlinear convergence was justified. See also [1] for the local analysis of the Adaptive cubic regularization methods.

Our paper is aimed to study local convergence of high-order methods, generalizing corresponding results from [22] in several ways. We establish local superlinear convergence of Tensor Method [18] of degree $p \ge 2$, in the case when the objective is composite, and its smooth part is uniformly convex of arbitrary degree *q* from the interval $2 \le q < p - 1$. For strongly convex functions ($q=2$), this gives the local convergence of degree *p*.

**Contents** We formulate our problem of interest and define a step of the Regularized Composite Tensor Method in Sect. 2. Then, we declare some of its properties, which are required for our analysis.

In Sect. 3, we prove local superlinear convergence of the Tensor Method in function value, and in the norm of minimal subgradient, under the assumption of uniform convexity of the objective.

In Sect. 4, we discuss global behavior of the method and justify sublinear and linear global rates of convergence for convex and uniformly convex cases, respectively.

One application of our developments is provided in Sect. 5. We show how local convergence can be applied for computing an inexact step in proximal methods. A global sublinear rate of convergence for the resulting scheme is also given.

**Notations and generalities** In what follows, we denote by $\mathbb {E}$ a finite-dimensional real vector space, and by $\mathbb {E}^*$ its dual spaced composed by linear functions on $\mathbb {E}$. For such a function $s \in \mathbb {E}^*$, we denote by $\langle s, x \rangle$ its value at $x \in \mathbb {E}$. Using a self-adjoint positive-definite operator $B: \mathbb {E}\rightarrow \mathbb {E}^*$ (notation $B = B^* \succ 0$), we can endow these spaces with mutually conjugate Euclidean norms:

$$\begin{aligned} \Vert x \Vert= & {} \langle B x, x \rangle ^{1/2}, \quad x \in \mathbb {E}, \quad \Vert g \Vert _* \; = \; \langle g, B^{-1} g \rangle ^{1/2}, \quad g \in \mathbb {E}^*. \end{aligned}$$

For a smooth function $f: \mathrm{dom} \,f \rightarrow \mathbb {R}$ with convex and open domain $\mathrm{dom} \,f \subseteq \mathbb {E}$, denote by $\nabla f(x)$ its gradient, and by $\nabla ^2 f(x)$ its Hessian evaluated at point $x \in \mathrm{dom} \,f \subseteq \mathbb {E}$. Note that

$$\begin{aligned} \nabla f(x)\in & {} \mathbb {E}^*, \quad \nabla ^2 f(x) h \; \in \; \mathbb {E}^*, \quad x \in \mathrm{dom} \,f, \; h \in \mathbb {E}. \end{aligned}$$

For non-differentiable convex function $f(\cdot )$, we denote by $\partial f(x) \subset \mathbb {E}^*$ its subdifferential at the point $x \in \mathrm{dom} \,f$.

In what follows, we often work with directional derivatives. For $p \ge 1$, denote by

$$\begin{aligned} D^p f(x)[h_1, \dots , h_p] \end{aligned}$$

the directional derivative of function *f* at *x* along directions $h_i \in \mathbb {E}$, $i = 1, \dots , p$. If all directions $h_1, \dots , h_p$ are the same, we apply a simpler notation

$$\begin{aligned} D^p f(x)[h]^p, \quad h \in \mathbb {E}. \end{aligned}$$

Note that $D^p f(x)[ \cdot ]$ is a *symmetric p-linear form*. Its *norm* is defined in the standard way:

1.1 $$\begin{aligned} \Vert D^pf(x) \Vert= & {} \max \limits _{h_1, \dots , h_p \in \mathbb {E}} \left\{ D^p f(x)[h_1, \dots , h_p ]: \; \Vert h_i \Vert \le 1, \, i = 1, \dots , p \right\} \nonumber \\= & {} \max \limits _{h \in \mathbb {E}} \left\{ \Big | D^p f(x)[h]^p\Big |: \; \Vert h \Vert \le 1 \right\} \end{aligned}$$

(for the last equation see, for example, Appendix 1 in [21]). Similarly, we define

1.2 $$\begin{aligned} \begin{array}{rcl} \Vert D^pf(x) - D^pf(y) \Vert= & {} \max \limits _{h \in \mathbb {E}} \left\{ \Big | D^p f(x)[h]^p - D^pf(y)[h]^p\Big |: \; \Vert h \Vert \le 1 \right\} . \end{array} \end{aligned}$$

In particular, for any $x \in \mathrm{dom} \,f$ and $h_1, h_2 \in \mathbb {E}$, we have

$$\begin{aligned} Df(x)[h_1]= & {} \langle \nabla f(x), h_1 \rangle , \quad D^2f(x)[h_1, h_2] \; = \; \langle \nabla ^2 f(x) h_1, h_2 \rangle . \end{aligned}$$

Thus, for the Hessian, our definition corresponds to a *spectral norm* of the self-adjoint linear operator (maximal module of all eigenvalues computed with respect to $B \succ 0$).

Finally, the Taylor approximation of function $f(\cdot )$ at $x \in \mathrm{dom} \,f$ is defined as follows:

$$\begin{aligned}&f(x+h) = \varOmega _p(f, x; x + h) + o(\Vert h\Vert ^p), \quad x+h \in \mathrm{dom} \,f,\\&\quad \varOmega _p(f,x;y) \; {\mathop {=}\limits ^{\mathrm {def}}}\; f(x) + \sum \limits _{k=1}^p {1 \over k!} D^k f(x)[y-x]^k, \quad y \in \mathbb {E}. \end{aligned}$$

Consequently, for all $y \in \mathbb {E}$ we have

1.3 $$\begin{aligned} \nabla \varOmega _p(f,x;y)= & {} \sum \limits _{k=1}^p {1 \over (k-1)!} D^k f(x)[y-x]^{k-1}, \end{aligned}$$

1.4 $$\begin{aligned} \nabla ^2 \varOmega _p(f,x;y)= & {} \sum \limits _{k=2}^p {1 \over (k-2)!} D^k f(x)[y-x]^{k-2}. \end{aligned}$$

## Main inequalities

In this paper, we consider the following *composite* convex minimization problem

2.1 $$\begin{aligned} \min \limits _{x \in \mathrm{dom} \,h} \Big \{ F(x) = f(x) + h(x) \Big \}, \end{aligned}$$

where $h: \mathbb {E}\rightarrow \mathbb {R}\cup \{+\infty \}$ is a *simple* proper closed convex function and $f \in C^{p,p}(\mathrm{dom} \,h)$ for a certain $p \ge 2$. In other words, we assume that the *p*th derivative of function *f* is Lipschitz continuous:

2.2 $$\begin{aligned} \begin{array}{rcl} \Vert D^p f(x) - D^p f(y) \Vert\le & {} L_p \Vert x - y \Vert , \quad x, y \in \mathrm{dom} \,h. \end{array} \end{aligned}$$

Assuming that $L_{p} < +\infty$, by the standard integration arguments we can bound the residual between function value and its Taylor approximation:

2.3 $$\begin{aligned} \begin{array}{rcl} | f(y) - \varOmega _p(f,x;y) |\le & {} {L_{p} \over (p+1)!} \Vert y - x \Vert ^{p+1}, \quad x, y \in \mathrm{dom} \,h. \end{array} \end{aligned}$$

Applying the same reasoning to functions $\langle \nabla f(\cdot ), h \rangle$ and $\langle \nabla ^2 f(\cdot ) h, h \rangle$ with direction $h \in \mathbb {E}$ being fixed, we get the following guarantees:

2.4 $$\begin{aligned} \Vert \nabla f(y) - \nabla \varOmega _p(f,x;y) \Vert _*\le & {} {L_p \over p!} \Vert y - x \Vert ^{p}, \end{aligned}$$

2.5 $$\begin{aligned} \Vert \nabla ^2 f(y) - \nabla ^2 \varOmega _p(f,x;y) \Vert\le & {} {L_p \over (p-1)!} \Vert y - x \Vert ^{p-1}, \end{aligned}$$

which are valid for all $x, y \in \mathrm{dom} \,h$.

Let us define now one step of the *Regularized Composite Tensor Method* (RCTM) of degree $p \ge 2$:

2.6 $$\begin{aligned} \begin{array}{rcl} T\equiv & {} T_H(x) \; {\mathop {=}\limits ^{\mathrm {def}}}\; \arg \min \limits _{y \in \mathbb {E}} \left\{ \varOmega _p(f,x;y) + {H \over (p+1)!} \Vert y - x \Vert ^{p+1} + h(y) \right\} . \end{array} \end{aligned}$$

It can be shown that for2.7the auxiliary optimization problem in (2.6) is *convex* (see Theorem 1 in [18]). This condition is crucial for implementability of our methods and we always assume it to be satisfied.

Let us write down the first-order optimality condition for the auxiliary optimization problem in (2.6):

2.8 $$\begin{aligned} \begin{array}{rcl} \langle \nabla \varOmega _p(f,x;T) + {H \over p!} \Vert T - x \Vert ^{p-1}B(T-x), y - T \rangle + h(y)\ge & {} h(T), \end{array} \end{aligned}$$

for all $y \in \mathrm{dom} \,h$. In other words, for vector

2.9 $$\begin{aligned} h'(T) \; {\mathop {=}\limits ^{\mathrm {def}}}\; - \left( \nabla \varOmega _p(f,x;T) + {H \over p!} \Vert T - x \Vert ^{p-1}B(T-x) \right) \end{aligned}$$

we have $h'(T) {\mathop {\in }\limits ^{(2.8)}} \partial h(T)$. This fact explains our notation

2.10 $$\begin{aligned} F'(T) \; {\mathop {=}\limits ^{\mathrm {def}}}\; \nabla f(T) + h'(T) \; \in \partial F(T). \end{aligned}$$

Let us present some properties of the point $T = T_H(x)$. First of all, we need some bounds for the norm of vector $F'(T)$. Note that

2.11 $$\begin{aligned} \Big \Vert F'(T) + {H \over p!} \Vert T - x \Vert ^{p-1}B(T-x) \Big \Vert _*&{{\mathop {=}\limits ^{(2.9)}}}&\Big \Vert \nabla f(T) - \nabla \varOmega _p(f,x;T) \Big \Vert _* \nonumber \\&{{\mathop {\le }\limits ^{(2.4)}}}&{L_p \over p!} \Vert T - x \Vert ^p. \end{aligned}$$

Consequently,

2.12 $$\begin{aligned} \begin{array}{rcl} \Vert F'(T) \Vert _*\le & {} {L_p+H \over p!} \Vert T - x \Vert ^p. \end{array} \end{aligned}$$

Secondly, we use the following lemma.

### Lemma 1

Let $\beta > 1$ and $H = \beta L_p$. Then

2.13 $$\begin{aligned} \begin{array}{rcl} \langle F'(T), x - T \rangle\ge & {} \left( {p! \over (p+1)L_p} \right) ^{1 \over p} \cdot \Vert F'(T) \Vert _*^{p+1 \over p} \cdot {(\beta ^2 - 1)^{p-1 \over 2p} \over \beta } \cdot {p \over (p^2-1)^{p-1 \over 2p}}. \end{array} \end{aligned}$$

In particular, if $\beta = p$, then

2.14 $$\begin{aligned} \begin{array}{rcl} \langle F'(T), x - T \rangle\ge & {} \left( {p! \over (p+1)L_p} \right) ^{1 \over p} \cdot \Vert F'(T) \Vert _*^{p+1 \over p}. \end{array} \end{aligned}$$

### Proof

Denote $r = \Vert T - x \Vert$, $h = {H \over p!}$, and $l = {L_p \over p!}$. Then inequality (2.11) can be written as follows:

$$\begin{aligned} \Vert F'(T) + h r^{p-1} B(T-x) \Vert ^2_*\le & {} l^2 r^{2p}. \end{aligned}$$

This means that

2.15 $$\begin{aligned} \begin{array}{rcl} \langle F'(T), x - T \rangle\ge & {} {1 \over 2 h r^{p-1}} \Vert F'(T) \Vert _*^2 + {r^{2p} (h^2 - l^2) \over 2h r^{p-1}}. \end{array} \end{aligned}$$

Denote

$$\begin{aligned} a= & {} {1 \over 2h} \Vert F'(T) \Vert _*^2, \quad b \; = \; {h^2 - l^2 \over 2h}, \quad \tau \; = \; r^{p-1}, \quad \alpha \; = \; {p+1 \over p-1}. \end{aligned}$$

Then inequality (2.15) can be rewritten as follows:

$$\begin{aligned} \langle F'(T) , x - T \rangle\ge & {} {a \over \tau } + b \tau ^{\alpha } \; \ge \; \min \limits _{t > 0} \left\{ {a \over t} + b t^{\alpha } \right\} \; = \; (1+\alpha ) \left( {a \over \alpha } \right) ^{\alpha \over 1 + \alpha } b^{1 \over 1 + \alpha }. \end{aligned}$$

Taking into account that $1+\alpha = {2p \over p-1}$ and ${\alpha \over 1 + \alpha } = {p + 1 \over 2p}$, and using the actual meaning of *a*, *b*, and $\alpha$, we get

$$\begin{aligned} \langle F'(T), x - T \rangle\ge & {} {2 p \over p-1} \cdot { \Vert F'(T) \Vert _*^{p+1 \over p} \over (2h)^{p+1 \over 2p}} \cdot {(p-1)^{p+1 \over 2p} \over (p+1)^{p+1 \over 2p}} \cdot {(h^2 - l^2)^{p-1 \over 2p} \over (2h)^{p-1 \over 2p}}\\= & {} \Vert F'(T) \Vert _*^{p+1 \over p} \cdot {(h^2 - l^2)^{p-1 \over 2p} \over h} \cdot {p \over (p+1)^{p+1 \over 2p} (p-1)^{p-1 \over 2p}}\\= & {} \Vert F'(T) \Vert _*^{p+1 \over p} \cdot {(h^2 - l^2)^{p-1 \over 2p} \over h} \cdot {p \over (p^2-1)^{p-1 \over 2p} (p+1)^{1 \over p}}. \end{aligned}$$

It remains to note that

$$\begin{aligned} {(h^2 - l^2)^{p-1 \over 2p} \over h}= & {} {(H^2 - L_p^2)^{p-1 \over 2p} \over H} \cdot (p!)^{1 \over p} \; = \; {(\beta ^2 - 1)^{p-1 \over 2p} \over \beta } \cdot \left( {p! \over L_p} \right) ^{1 \over p}. \end{aligned}$$

$$\square $$

## Local convergence

The main goal of this paper consists in analyzing the local behavior of the *Regularized Composite Tensor Method* (RCTM):

3.1 $$\begin{aligned} \begin{array}{rcl} x_0 \; \in \; \mathrm{dom} \,h, \quad x_{k+1}= & {} T_H(x_k), \quad k \ge 0, \end{array} \end{aligned}$$

as applied to the problem (2.1). In order to prove local superlinear convergence of this scheme, we need one more assumption.

### Assumption 1

The objective in problem (2.1) is uniformly convex of degree $q \ge 2$. Thus, for all $x, y \in \mathrm{dom} \,h$ and for all $G_x \in \partial F(x), G_y \in \partial F(y)$, it holds:

3.2 $$\begin{aligned} \begin{array}{rcl} \langle G_x - G_y, x - y \rangle\ge & {} \sigma _q \Vert x - y \Vert ^q, \end{array} \end{aligned}$$

for certain $$\sigma _q > 0$$.

It is well known that this assumption guarantees the uniform convexity of the objective function (see, for example, Lemma 4.2.1 in [19]):

3.3 $$\begin{aligned} \begin{array}{rcl} F(y)\ge & {} F(x) + \langle G_x, y - x \rangle + {\sigma _q \over q} \Vert y - x \Vert ^q, \quad y \in \mathrm{dom} \,h, \end{array} \end{aligned}$$

where $G_x$ is an arbitrary subgradient from $\partial F(x)$. Therefore,

3.4 $$\begin{aligned} F^*= & {} \min \limits _{y \in \mathrm{dom} \,h} F(y) \; \ge \; \min \limits _{y \in \mathbb {E}} \left\{ F(x) + \langle G_x, y - x \rangle + {\sigma _q \over q} \Vert y - x \Vert ^q \right\} \nonumber \\= & {} F(x) - {q-1 \over q} \left( {1 \over \sigma _q} \right) ^{1 \over q-1} \Vert G_x \Vert _*^{q \over q-1}. \end{aligned}$$

This simple inequality gives us the following local convergence rate for RCTM.

### Theorem 1

For any $k \ge 0$ we have

3.5 $$\begin{aligned} \begin{array}{rcl} F(x_{k+1}) - F^*\le & {} (q-1) q^{p-q+1 \over q-1} \bigl ({1 \over \sigma _q}\bigr )^{p+1 \over q-1} \left( {L_p + H \over p!} \right) ^{q \over q - 1} \big [F(x_k) - F^* \big ]^{p \over q-1}. \end{array} \end{aligned}$$

### Proof

Indeed, for any $k \ge 0$ we have

$$\begin{aligned}&F(x_k) - F^* \ge F(x_k) - F(x_{k+1}) \\&\quad {{\mathop {\ge }\limits ^{(3.3)}}} \; \langle F'(x_{k+1}), x_k - x_{k+1} \rangle + {\sigma _q \over q} \Vert x_k - x_{k+1} \Vert ^q\\&\quad {{\mathop {\ge }\limits ^{(2.13)}}} \; {\sigma _q \over q} \Vert x_k - x_{k+1} \Vert ^q \; {{\mathop {\ge }\limits ^{(2.12)}}} \; {\sigma _q \over q} \left( {p! \over L_p+H} \Vert F'(x_{k+1}) \Vert _* \right) ^{q \over p}\\&\quad {{\mathop {\ge }\limits ^{(3.4)}}} \; {\sigma _q \over q} \left( {p! \over L_p+H}\right) ^{q \over p} \left( {q \, \sigma _q^{1 \over q-1} \over q-1} (F(x_{k+1})-F^*) \right) ^{q -1\over p}. \end{aligned}$$

And this is exactly inequality (3.5). $\square$

### Corollary 1

If $p > q-1$, then method (3.1) has local superlinear rate of convergence for problem (2.1).

### Proof

Indeed, in this case ${p \over q-1} > 1$. $\square$

For example, if $q = 2$ (strongly convex function) and $p=2$ (Cubic Regularization of the Newton Method), then the rate of convergence is quadratic. If $q=2$, and $p = 3$, then the local rate of convergence is cubic, etc.

Let us study now the local convergence of the method (3.1) in terms of the norm of gradient. For any $x \in \mathrm{dom} \,h$ denote

3.6 $$\begin{aligned} \eta (x) \; {\mathop {=}\limits ^{\mathrm {def}}}\; \min \limits _{g \in \partial h(x)} \Vert \nabla f(x) + g \Vert _*. \end{aligned}$$

If $\partial h(x) = \emptyset$, we set $\eta (x) = +\infty$.

### Theorem 2

For any $k \ge 0$ we have

3.7 $$\begin{aligned} \begin{array}{rcl} \eta (x_{k+1})&\, \le \,&\Vert F'(x_{k + 1}) \Vert _{*} \; \le \; {L_p + H \over p!} \left[ {1 \over \sigma _q} \, \eta (x_k) \right] ^{p \over q-1}. \end{array} \end{aligned}$$

### Proof

Indeed, in view of inequality (3.2), we have

$$\begin{aligned} \langle \nabla f(x_k) + g_k, x_{k} - x_{k + 1} \rangle\ge & {} \langle F'(x_{k + 1}), x_k - x_{k + 1} \rangle + \sigma _q \Vert x_k - x_{k + 1}\Vert ^q \\&{{\mathop {\ge }\limits ^{(2.13)}}}&\sigma _q \Vert x_k - x_{k + 1}\Vert ^{q}, \end{aligned}$$

where $g_k$ is an arbitrary vector from $\partial h(x_k)$. Therefore, we conclude that

$$\begin{aligned} \eta (x_k)\ge & {} \sigma _q \Vert x_k - x_{k+1} \Vert ^{q-1}. \end{aligned}$$

It remains to use inequality (2.12). $\square$

As we can see, the condition for superlinear convergence of the method (3.1) in terms of the norm of the gradient is the same as in Corollary 1: we need to have ${p \over q-1} > 1$, that is $p > q-1$. Moreover, the local rate of convergence has the same order as that for the residual of the function value.

According to Theorem 1, the region of superlinear convergence of RCTM in terms of the function value is as follows:

3.8 $$\begin{aligned} \begin{array}{rcl} \mathcal {Q}= & {} \left\{ x \in \mathrm{dom} \,h: \; F(x) - F^* \; \le \; {1 \over q} \cdot \biggl ( { \sigma _q^{p + 1} \over (q - 1)^{q - 1} } \cdot \Bigl ( { p! \over L_p + H } \Bigr )^{q} \biggr )^{1 \over p - q + 1} \right\} . \end{array} \end{aligned}$$

Alternatively, by Theorem 2, in terms of the norm of minimal subgradient (3.6), the region of superlinear convergence looks as follows:

3.9 $$\begin{aligned} \begin{array}{rcl} \mathcal {G}= & {} \left\{ x \in \mathrm{dom} \,h: \; \eta (x) \; \le \; \biggl ( \sigma _q^{p} \cdot \Bigl ( { p! \over L_p + H } \Bigr )^{q - 1} \biggr )^{1 \over p - q + 1} \right\} . \end{array} \end{aligned}$$

Note that these sets can be very different. Indeed, set $\mathcal {Q}$ is a closed and convex neighborhood of the point $x^*$. At the same time, the structure of the set $\mathcal {G}$ can be very complex since in general the function $\eta (x)$ is discontinuous. Let us look at simple example where $h(x) = \text{ Ind}_Q(x)$, the indicator function of a closed convex set *Q*.

### Example 1

Consider the following optimization problem:

3.10 $$\begin{aligned} \min \limits _{x \in \mathbb {R}^2} \left\{ f(x) : \; \Vert x \Vert ^2 \; {\mathop {=}\limits ^{\mathrm {def}}}\; (x^{(1)})^2 + (x^{(2)})^2 \le 1 \right\} , \end{aligned}$$

with

$$\begin{aligned} f(x)= & {} \frac{\sigma _2}{2}\Vert x - \bar{x}\Vert ^2 + \frac{2 \sigma _3}{3}\Vert x - \bar{x}\Vert ^3, \end{aligned}$$

for some fixed $\sigma _2, \sigma _3 > 0$ and $\bar{x} = (0, -2) \in \mathbb {R}^2$. We have

$$\begin{aligned} \nabla f(x) = r(x) \cdot ( x^{(1)}, x^{(2)} + 2), \end{aligned}$$

where $$r: \mathbb {R}^2 \rightarrow \mathbb {R}$$ is

$$\begin{aligned} r(x) = \sigma _2 + 2\sigma _3 \Vert x - \bar{x}\Vert . \end{aligned}$$

Note that *f* is uniformly convex of degree $q = 2$ with constant $\sigma _2$, and for $q = 3$ with constant $\sigma _3$ (see Lemma 4.2.3 in [19]). Moreover, we have for any $\nu \in [0, 1]$:

$$\begin{aligned} \langle \nabla f(x) - \nabla f(y), x - y \rangle\ge & {} \sigma _2 \Vert x - y\Vert ^2 + \sigma _3 \Vert x - y\Vert ^3 \\\ge & {} \min \limits _{t \ge 0} \Bigl \{ \frac{\sigma _2}{t^{\nu }} + \sigma _3 t^{1 - \nu } \Bigr \} \cdot \Vert x - y\Vert ^{2 + \nu } \\\ge & {} \sigma _2^{1 - \nu } \sigma _3^{\nu } \cdot \Vert x - y\Vert ^{2 + \nu }. \end{aligned}$$

Hence, this function is uniformly convex of any degree $q \in [2, 3]$. At the same time, the Hessian of *f* is Lipschitz continuous with constant $L_2 = 4 \sigma _3$ (see Lemma 4.2.4 in [19]).

Clearly, in this problem $x^*=(0,-1)$, and it can be written in the composite form (2.1) with

$$\begin{aligned} h(x)= & {} \left\{ \begin{array}{ll} + \infty , &{}\quad \text{ if } \Vert x \Vert > 1, \\ 0, &{}\quad \text{ otherwise. } \end{array} \right. \end{aligned}$$

Note that for $x \in \mathrm{dom} \,h \equiv \{ x: \; \Vert x \Vert \le 1\}$, we have

$$\begin{aligned} \partial h(x) \; = \; \left\{ \begin{array}{ll} 0, &{}\quad \text{ if } \Vert x \Vert < 1, \\ \{ \gamma x, \, \gamma \ge 0 \}, &{}\quad \text{ if } \Vert x \Vert = 1. \end{array} \right. \end{aligned}$$

Therefore, if $\Vert x \Vert < 1$, then $\eta (x) = \Vert \nabla f(x) \Vert \ge \sigma _2$. If $\Vert x \Vert = 1$, then

$$\begin{aligned} \eta ^2(x)&{{\mathop {=}\limits ^{(3.6)}}}&\min \limits _{\gamma \ge 0} \Bigl \{ \bigl [ (r(x) + \gamma ) x^{(1)} \bigr ]^2 + \bigl [ (r(x) + \gamma ) x^{(2)} + 2 r(x) \bigr ]^2 \Bigr \} \\= & {} \min \limits _{\gamma \ge 0} \Bigl \{ (r(x) + \gamma )^2 + 4r(x) (r(x) + \gamma ) x^{(2)} + 4 r^2(x) \Bigr \} \\= & {} \left\{ \begin{array}{ll} 4r^2(x) (1 - (x^{(2)})^2), &{}\quad \text{ if } x^{(2)} \le -\frac{1}{2}, \\ r^2(x) (5 + 4 x^{(2)}), &{}\quad \text{ otherwise. } \end{array} \right. \end{aligned}$$

Thus, in any neighbourhood of $x^*$, $\eta (x)$ vanishes only along the boundary of the feasible set. $\square$

So, the question arises how the Tensor Method (3.1) could come to the region $\mathcal {G}$. The answer follows from the inequalities derived in Sect.  2. Indeed,

$$\begin{aligned} \Vert F'(x_{k+1}) \Vert _* \; {{\mathop {\le }\limits ^{(2.12)}}} \; {L_p + H \over p!} \Vert x_k - x_{k+1} \Vert ^p, \end{aligned}$$

and

$$\begin{aligned} F(x_k) - F(x_{k+1})\ge & {} \langle F'(x_{k+1}), x_k - x_{k+1} \rangle \\&{{\mathop {\ge }\limits ^{(2.14)}}}&\left( {p! \over (p+1)L_p} \right) ^{1 \over p} \cdot \Vert F'(x_{k+1}) \Vert ^{p+1 \over p}_*. \end{aligned}$$

Thus, at some moment the norm $\Vert F'(x_k) \Vert _*$ will be small enough to enter $\mathcal {G}$.

## Global complexity bounds

Let us briefly discuss the global complexity bounds of the method (3.1), namely the number of iterations required for coming from an arbitrary initial point $x_0 \in \mathrm{dom} \,h$ to the region $\mathcal {Q}$. First, note that for every step $T = T_H(x)$ of the method with parameter $H \ge p L_p$, we have

$$\begin{aligned} F(T)&{{\mathop {\le }\limits ^{(2.3)}}}&\varOmega _p(f,x;T) + \frac{H}{(p + 1)!}\Vert T - x\Vert ^{p + 1} + h(T) \\&{{\mathop {=}\limits ^{(2.6)}}}&\min \limits _{y \in \mathbb {E}} \Bigl \{ \varOmega _p(f,x;y) + \frac{H}{(p + 1)!}\Vert y - x\Vert ^{p + 1} + h(y) \Bigr \} \\&{{\mathop {\le }\limits ^{(2.3)}}}&\min \limits _{y \in \mathbb {E}} \Bigl \{ F(y) + \frac{H + L_p}{(p + 1)!} \Vert y - x\Vert ^{p + 1} \Bigr \}. \end{aligned}$$

Therefore,

4.1 $$\begin{aligned} \begin{array}{rcl} F(T(x)) - F^{*}\le & {} \frac{H + L_p}{(p + 1)!}\Vert x - x^{*}\Vert ^{p + 1}, \quad x \in \mathrm{dom} \,h, \end{array} \end{aligned}$$

with $x^{*} {\mathop {=}\limits ^{\mathrm {def}}}\arg \min \limits _{y \in \mathbb {E}} F(y)$, which exists by our assumption. Denote by *D* the maximal radius of the initial level set of the objective, which we assume to be finite:

$$\begin{aligned} \begin{array}{rcl} D \;\; {\mathop {=}\limits ^{\mathrm {def}}}\; \sup \limits _{x \in \mathrm{dom} \,h} \Bigl \{ \Vert x - x^{*}\Vert :\; F(x) \le F(x_0) \Bigr \} \;< & {} \; +\infty . \end{array} \end{aligned}$$

Then, by monotonicity of the method (3.1) and by convexity we conclude

4.2 $$\begin{aligned} {1 \over D}\Bigl ( F(x_{k + 1}) - F^* \Bigr ) \; \le \; {1 \over D}\langle F'(x_{k + 1}), x_{k + 1} - x^{*} \rangle \; \le \; \Vert F'(x_{k + 1})\Vert _{*}. \end{aligned}$$

In the general convex case, we can prove the global sublinear rate of convergence of the Tensor Method of the order $O({1 / k^p})$ [18]. For completeness of presentation, let us prove an extension of this result onto the composite case.

### Theorem 3

For the method (3.1) with $H = pL_p$ we have

4.3 $$\begin{aligned} \begin{array}{rcl} F(x_{k}) - F^{*}\le & {} { (p + 1) (2p)^p \over p! } \cdot {L_p D^{p + 1} \over (k - 1)^p}, \qquad k \ge 2. \end{array} \end{aligned}$$

### Proof

Indeed, in view of (2.14) and (4.2), we have for every $k \ge 0$

$$\begin{aligned} F(x_{k}) - F(x_{k + 1})\ge & {} \langle F'(x_{k + 1}), x_k - x_{k + 1} \rangle \\&{{\mathop {\ge }\limits ^{(2.14)}}}&\left( {p! \over (p+1)L_p} \right) ^{1 \over p} \cdot \Vert F'(x_{k + 1}) \Vert _*^{p+1 \over p} \\&{{\mathop {\ge }\limits ^{(4.2)}}}&\left( {p! \over (p+1)L_p D^{p + 1} } \right) ^{1 \over p} \cdot \Bigl ( F(x_{k + 1}) - F^* \Bigr )^{ p + 1 \over p }. \end{aligned}$$

Denoting $\delta _k = F(x_k) - F^*$ and $C = \left( {p! \over (p+1) L_p D^{p + 1} }\right) ^{1 \over p}$, we obtain the following recurrence:

4.4 $$\begin{aligned} \begin{array}{rcl} \delta _{k} - \delta _{k + 1}\ge & {} C \delta _{k + 1}^{p + 1 \over p}, \qquad k \ge 0, \end{array} \end{aligned}$$

or for $\mu _k = C^p \delta _k {{\mathop {\le }\limits ^{(4.1)}}} 1$, as follows:

$$\begin{aligned} \mu _{k} - \mu _{k + 1}\ge & {} \mu _{k + 1}^{p + 1 \over p}, \qquad k \ge 0. \end{aligned}$$

Then, Lemma 1.1 from [8] provides us with the following guarantee:

$$\begin{aligned} \mu _{k}\le & {} \Bigl ( \frac{p(1 + \mu _1^{1 / p})}{k - 1} \Bigr )^p \; \le \; \Bigl ( \frac{2p}{k - 1} \Bigr )^p, \quad k \ge 2. \end{aligned}$$

Therefore,

$$\begin{aligned} \delta _k= & {} {\mu _{k} \over C^p} \; \le \; \left( { 2p \over C (k - 1) }\right) ^p \; = \; { (p + 1) (2p)^p \over p! } \cdot {L_p D^{p + 1} \over (k - 1)^p}, \qquad k \ge 2. \end{aligned}$$

$$\square $$

For a given degree $q \ge 2$ of uniform convexity with $\sigma _q > 0$, and for RCTM of order $p \ge q - 1$, let us denote by $\omega _{p, q}$ the following *condition number*:

$$\begin{aligned} \omega _{p, q} \; {\mathop {=}\limits ^{\mathrm {def}}}\; \frac{p + 1}{p!} \cdot \Bigl ( \frac{q - 1}{q} \Bigr )^{q - 1} \cdot \frac{L_p D^{p - q + 1}}{\sigma _q}. \end{aligned}$$

### Corollary 2

In order to achieve the region $\mathcal {Q}$ it is enough to perform

4.5 $$\begin{aligned} \Biggl \lceil 2p \cdot \biggl ( { q^{q} \over (q - 1)^{q - 1} } \cdot \omega _{p, q}^{\frac{p + 1}{p}} \biggr )^{1 \over p - q + 1} \Biggr \rceil + 2 \end{aligned}$$

iterations of the method.

### Proof

Plugging (3.8) into (4.3). $$\square $$

We can improve this estimate, knowing that the objective is globally uniformly convex (3.2). Then the linear rate of convergence arises at the first state, till the entering in the region $\mathcal {Q}$.

### Theorem 4

Let $\sigma _q > 0$ with $q \le p + 1$. Then for the method (3.1) with $H = pL_p$, we have

4.6 $$\begin{aligned} \begin{array}{rcl} F(x_{k}) - F^{*}\le & {} \exp \left( -{k \over 1 + \omega ^{1/p}_{p, q}} \right) \cdot \bigl ( F(x_{0}) - F^*\bigr ), \qquad k \ge 1. \end{array} \end{aligned}$$

Therefore, for a given $\varepsilon > 0$ to achieve $F(x_K) - F^{*} \le \varepsilon$, it is enough to set

4.7 $$\begin{aligned} \begin{array}{rcl} K= & {} \left\lceil (1+\omega ^{1/p}_{p,q}) \cdot \log {\frac{F(x_0) - F^{*}}{\varepsilon }} \right\rceil + 1. \end{array} \end{aligned}$$

### Proof

Indeed, for every $k \ge 0$

$$\begin{aligned} F(x_{k}) - F(x_{k + 1})\ge & {} \langle F'(x_{k + 1}), x_k - x_{k + 1} \rangle \\&{{\mathop {\ge }\limits ^{(2.14)}}}&\left( {p! \over (p+1)L_p} \right) ^{1 \over p} \cdot \Vert F'(x_{k + 1}) \Vert _*^{p+1 \over p} \\= & {} \left( {p! \over (p+1)L_p} \right) ^{1 \over p} \cdot \Vert F'(x_{k + 1}) \Vert _*^{p - q + 1 \over p} \cdot \Vert F'(x_{k + 1}) \Vert _*^{q \over p} \\&{\mathop {\!}\limits ^{(4.2),(3.4)}}\!{\ge }\!\!&\left( {p! \over p \!+\! 1} \cdot { \sigma _q \over L_p D^{p \!-\! q\! +\! 1}} \right) ^{1 \over p} \cdot \left( { q \over q \!-\! 1 }\right) ^{q \!-\! 1 \over p} \cdot \Bigl ( F(x_{k \!+\! 1})\! -\! F^* \Bigr ) \\= & {} \left( \frac{1}{\omega _{p, q}} \right) ^{1 \over p} \cdot \Bigl ( F(x_{k + 1}) - F^* \Bigr ). \end{aligned}$$

Denoting $\delta _k = F(x_k) - F^{*}$, we obtain

$$\begin{aligned} \delta _{k + 1}\le & {} {\omega ^{1/p}_{p,q} \over 1 + \omega ^{1/p}_{p,q}} \cdot \delta _k \; \le \; \exp \left( - {1 \over 1 + \omega ^{1/p}_{p,q}} \right) \cdot \delta _k, \qquad k \ge 1. \end{aligned}$$

$$\square $$

We see that, for RCTM with $p \ge 2$ minimizing the uniformly convex objective of degree $q \le p + 1$, the condition number $\omega ^{1/p}_{p, q}$ is the main factor in the global complexity estimates (4.5) and (4.7). Since in general this number may be arbitrarily big, complexity estimate $\tilde{O}(\omega _{p, q}^{1 / p})$ in (4.7) is much better than the estimate $O(\omega _{p, q}^{(p + 1) / (p(p - q + 1))})$ in (4.5) because of relation ${ p + 1 \over p - q + 1} \ge 1$.

These global bounds can be improved, by using the *universal* [3, 10] and the *accelerated* [7, 9, 10, 17, 28] high-order schemes.

High-order tensor methods for minimizing the gradient norm were developed in [4]. These methods achieve near-optimal global convergence rates, and can be used for coming into the region $\mathcal {G}$ (3.9). Note, that for the composite minimization problems, some modification of these methods is required, which ensures minimization of the *subgradient* norm.

Finally, let us mention some recent results [12, 20], where it was shown that a proper implementation of the third-order schemes by second-order oracle may lead to a significant acceleration of the methods. However, the relation of these techniques to the local convergence needs further investigations.

## Application to proximal methods

Let us discuss now a general approach, which uses the local convergence of the methods for justifying the global performance of proximal iterations.

The proximal method [23] is one of the classical methods in theoretical optimization. Every step of the method for solving problem (2.1) is a minimization of the regularized objective:

5.1 $$\begin{aligned} \begin{array}{rcl} x_{k + 1}= & {} \arg \min \limits _{x \in \mathbb {E}} \Bigl \{ a_{k + 1} F(x) + \frac{1}{2}\Vert x - x_k\Vert ^2 \Bigr \}, \qquad k \ge 0, \end{array} \end{aligned}$$

where $\{ a_k \}_{k \ge 1}$ is a sequence of positive coefficients, related to the iteration counter.

Of course, in general, we can hope only to solve subproblem (5.1) inexactly. The questions of practical implementations and possible generalizations of the proximal method, are still in the area of intensive research (see, for example [11, 25–27]).

One simple observation on the subproblem (5.1) is that it is 1-strongly convex. Therefore, if we would be able to pick an initial point from the region of superlinear convergence (3.8) or (3.9), we could minimize it very quickly by RCTM of degree $p \ge 2$ up to arbitrary accuracy. In this section, we are going to investigate this approach. For the resulting scheme, we will prove the global rate of convergence of the order $\tilde{O}(1 / k^{p + 1 \over 2})$.

Denote by $\varPhi _{k + 1}$ the regularized objective from (5.1):

$$\begin{aligned} \varPhi _{k + 1}(x) \; {\mathop {=}\limits ^{\mathrm {def}}}\; a_{k + 1} F(x) + \frac{1}{2}\Vert x - x_k\Vert ^2 \; = \; a_{k + 1} f(x) + \frac{1}{2}\Vert x - x_k\Vert ^2 + a_{k + 1} h(x). \end{aligned}$$

We fix a sequences of accuracies $\{\delta _k\}_{k \ge 1}$ and relax the assumption on exact minimization in (5.1). Now, at every step we need to find a point $x_{k + 1}$ and corresponding subgradient vector $g_{k + 1} \in \partial \varPhi _{k + 1}(x_{k + 1})$ with bounded norm:

5.2 $$\begin{aligned} \begin{array}{rcl} \Vert g_{k + 1}\Vert _{*}\le & {} \delta _{k + 1}. \end{array} \end{aligned}$$

Denote

$$\begin{aligned} F'(x_{k + 1}) \; {\mathop {=}\limits ^{\mathrm {def}}}\; \frac{1}{a_{k + 1}}( g_{k + 1} - B(x_{k + 1} - x_k)) \; \in \; \partial F(x_{k + 1}). \end{aligned}$$

The following global convergence result holds for the general proximal method with inexact minimization criterion (5.2).

### Theorem 5

Assume that there exist a minimum $x^{*} \in \mathrm{dom} \,h$ of the problem (2.1). Then, for any $k \ge 1$, we have

5.3 $$\begin{aligned} \begin{array}{rcl} \sum \limits _{i = 1}^k a_i(F(x_i) - F^{*}) + \frac{1}{2}\sum \limits _{i = 1}^k a_i^2 \Vert F'(x_i)\Vert _{*}^2 + \frac{1}{2}\Vert x_k - x^{*}\Vert ^2\le & {} R_k(\delta ), \end{array} \end{aligned}$$

where

$$\begin{aligned} R_k(\delta ) \; {\mathop {=}\limits ^{\mathrm {def}}}\; \frac{1}{2}\left( \Vert x_0 - x^{*}\Vert + \sum \limits _{i = 1}^k \delta _i \right) ^2. \end{aligned}$$

### Proof

First, let us prove that for all $k \ge 0$ and for every $x \in \mathrm{dom} \,h$, we have

5.4 $$\begin{aligned} \begin{array}{rcl} \frac{1}{2}\Vert x_0 - x\Vert ^2 + \sum \limits _{i = 1}^k a_i F(x)\ge & {} \frac{1}{2}\Vert x_k - x\Vert ^2 + C_k(x), \end{array} \end{aligned}$$

where

$$\begin{aligned} C_k(x) \; {\mathop {=}\limits ^{\mathrm {def}}}\; \sum \limits _{i = 1}^k \left( a_i F(x_i) + \frac{a_i^2}{2} \Vert F'(x_i)\Vert _{*}^2 + \langle g_i, x - x_{i - 1} \rangle - \frac{\delta _i^2}{2} \right) . \end{aligned}$$

This is obviously true for $k = 0$. Let it hold for some $k \ge 0$. Consider the step number $k + 1$ of the inexact proximal method.

By condition (5.2), we have

$$\begin{aligned} \Vert a_{k + 1} F'(x_{k + 1}) + B(x_{k + 1} - x_k) \Vert _{*}^2\le & {} \delta _{k + 1}^2. \end{aligned}$$

Equivalently,

5.5 $$\begin{aligned} \langle a_{k + 1} F'(x_{k + 1}), x_k - x_{k + 1} \rangle \ge \frac{a_{k + 1}^2}{2}\Vert F'(x_{k + 1})\Vert _{*}^2 + \frac{1}{2}\Vert x_{k + 1} - x_k\Vert ^2 - \frac{\delta _{k + 1}^2}{2}.\nonumber \\ \end{aligned}$$

Therefore, using the inductive assumption and strong convexity of $\varPhi _{k + 1}(\cdot )$, we conclude

$$\begin{aligned}&\frac{1}{2}\Vert x_0 - x\Vert ^2 + \sum \limits _{i = 1}^{k + 1} a_i F(x) \; = \; \frac{1}{2}\Vert x_0 - x\Vert ^2 + \sum \limits _{i = 1}^k a_i F(x) + a_{k + 1} F(x) \\&\quad \; {{\mathop {\ge }\limits ^{(5.4)}}} \; \varPhi _{k + 1}(x) + C_k(x) \\&\quad \ge \;\;\, \varPhi _{k + 1}(x_{k + 1}) + \langle g_{k + 1}, x - x_{k + 1} \rangle + \frac{1}{2}\Vert x_{k + 1} - x\Vert ^2 + C_k(x) \\&\quad = \;\;\, a_{k + 1} F(x_{k + 1}) + \frac{1}{2}\Vert x_{k + 1} - x_k\Vert ^2 + \langle g_{k + 1}, x_k - x_{k + 1} \rangle \\&\qquad + \;\;\, \langle g_{k + 1}, x - x_k \rangle + \frac{1}{2}\Vert x_{k + 1} - x\Vert ^2 + C_k(x) \\&\quad = \;\;\, a_{k + 1} F(x_{k + 1}) + \langle a_{k + 1} F'(x_{k + 1}), x_k - x_{k + 1} \rangle - \frac{1}{2}\Vert x_{k + 1} - x_k\Vert ^2 \\&\qquad + \;\;\, \langle g_{k + 1}, x - x_k \rangle + \frac{1}{2}\Vert x_{k + 1} - x\Vert ^2 + C_k(x) \\&\quad {{\mathop {\ge }\limits ^{(5.5)}}} \; a_{k + 1} F(x_{k + 1}) + \frac{a_{k + 1}^2}{2}\Vert F'(x_{k + 1})\Vert _{*}^2 - \frac{\delta _{k + 1}^2}{2} \\&\qquad + \;\;\, \langle g_{k + 1}, x - x_k \rangle + \frac{1}{2}\Vert x_{k + 1} - x\Vert ^2 + C_k(x) \\&\quad = \;\;\, \frac{1}{2}\Vert x_{k + 1} - x\Vert ^2 + C_{k + 1}(x). \end{aligned}$$

Thus, inequality (5.4) is valid for all $k \ge 0$.

Now, plugging $x \equiv x^{*}$ into (5.4), we have

5.6 $$\begin{aligned}&\sum \limits _{i = 1}^k a_i (F(x_i) - F^{*}) + \frac{1}{2}\sum \limits _{i = 1}^k a_i^2 \Vert F'(x_i)\Vert _{*}^2 + \frac{1}{2}\Vert x_k - x^{*}\Vert ^2 \nonumber \\&\quad \le \;\;\, \frac{1}{2}\Vert x_0 - x^{*}\Vert ^2 + \frac{1}{2}\sum \limits _{i = 1}^k \delta _i^2 + \sum \limits _{i = 1}^k \langle g_i, x_{i - 1} - x^{*} \rangle \nonumber \\&\quad {{\mathop {\le }\limits ^{(5.2)}}} \; \frac{1}{2}\Vert x_0 - x^{*}\Vert ^2 + \frac{1}{2}\sum \limits _{i = 1}^k \delta _i^2 + \sum \limits _{i = 1}^k \delta _i \Vert x_{i - 1} - x^{*} \Vert {\mathop {=}\limits ^{\mathrm {def}}}\alpha _k. \end{aligned}$$

In order to finish the proof, it is enough to show that $\alpha _k \le R_k(\delta )$.

Indeed,

$$\begin{aligned} \alpha _{k + 1}= & {} \alpha _k + \frac{1}{2} \delta _{k + 1}^2 + \delta _{k + 1} \Vert x_k - x^{*}\Vert \\&{{\mathop {\le }\limits ^{(5.6)}}}&\alpha _k + \frac{1}{2}\delta _{k + 1}^2 + \delta _{k + 1} \sqrt{2 \alpha _k} \\= & {} \left( \sqrt{\alpha _k} + \frac{1}{\sqrt{2}}\delta _{k + 1} \right) ^2. \end{aligned}$$

Therefore,

$$\begin{aligned} \sqrt{\alpha _k}\le & {} \sqrt{\alpha _{k - 1}} + \frac{1}{\sqrt{2}}\delta _{k} \; \le \; \cdots \; \le \; \sqrt{\alpha _0} + \frac{1}{\sqrt{2}}\sum \limits _{i = 1}^k \delta _i \\= & {} \frac{1}{\sqrt{2}}\left( \Vert x_0 - x^{*}\Vert + \sum \limits _{i = 1}^k \delta _i \right) \; = \; \sqrt{R_k(\delta )}. \end{aligned}$$

$$\square $$

Now, we are ready to use the result on the local superlinear convergence of RCTM in the norm of subgradient (Theorem 2), in order to minimize $\varPhi _{k + 1}(\cdot )$ at every step of inexact proximal method.

Note that

$$\begin{aligned} \partial \varPhi _{k + 1}(x)= & {} a_{k + 1} \partial F(x) + B(x - x_k), \end{aligned}$$

and it is natural to start minimization process from the previous point $x_k$, for which $\partial \varPhi _{k + 1}(x_k) = a_{k + 1} \partial F(x_k)$. Let us also notice, that the Lipschitz constant of the *p*th derivative ($p \ge 2$) of the smooth part of $\varPhi _{k + 1}$ is $a_{k + 1} L_p$.

Using our previous notation, one step of RCTM can be written as follows:

$$\begin{aligned}&T_H(\varPhi _{k + 1}, z) \\&\;\;\, {\mathop {=}\limits ^{\mathrm {def}}}\;\;\, \arg \min \limits _{y \in \mathbb {E}} \Bigl \{ a_{k \!+\! 1} \varOmega _{p}(f, z; y) + \frac{H}{(p \!+\! 1)!}\Vert y - z\Vert ^{p \!+\! 1} \!+\! a_{k \!+\! 1}h(y) + \frac{1}{2}\Vert y - x_k\Vert ^2 \Bigr \}, \end{aligned}$$

where $H = a_{k + 1}pL_p$. Then, a sufficient condition for $z = x_k$ to be in the region of superlinear convergence (3.9) is

$$\begin{aligned} a_{k + 1} \Vert F'(x_k) \Vert _*\le & {} \left( p! \over a_{k + 1} (p + 1) L_p \right) ^{1 \over p - 1}, \end{aligned}$$

or, equivalently

$$\begin{aligned} a_{k + 1}\le & {} \left( {1 \over \Vert F'(x_k)\Vert _{*} }\right) ^{p - 1 \over p} \left( { p! \over (p + 1) L_p }\right) ^{1 \over p}. \end{aligned}$$

To be sure that $x_k$ is strictly inside the region, we can pick:

5.7 $$\begin{aligned} \boxed { \begin{array}{rcl} a_{k + 1}= & {} \left( {1 \over 2 \Vert F'(x_k)\Vert _{*}} \right) ^{p - 1 \over p} \left( p! \over (p + 1) L_p \right) ^{1 \over p} \end{array} } \end{aligned}$$

Note, that this rule requires fixing an initial subgradient $F'(x_0) \in \partial F(x_0)$, in order to choose $a_1$.

Finally, we apply the following steps:

5.8 $$\begin{aligned} \begin{array}{rcl} z_0 \; = \; x_k, \quad z_{t+1}= & {} T_{H}(\varPhi _{k + 1}, z_t), \quad t \ge 0. \end{array} \end{aligned}$$

We can estimate the required number of these iterations as follows.

### Lemma 2

At every iteration $k \ge 0$ of the inexact proximal method, in order to achieve $\Vert \varPhi '_{k + 1}(z_t) \Vert _{*} \le \delta _{k + 1}$, it is enough to perform

5.9 $$\begin{aligned} \begin{array}{rcl} t_k= & {} \biggl \lceil \frac{1}{\log _2 p} \cdot \log _2 \log _2 \left( \frac{2 D_k(\delta ) }{ \delta _{k + 1}} \right) \biggr \rceil \end{array} \end{aligned}$$

steps of RCTM (5.8), where

$$\begin{aligned} D_k(\delta ) \; {\mathop {=}\limits ^{\mathrm {def}}}\; \max \biggl \{ \Vert x_0 - x^{*}\Vert + \sum \limits _{i = 1}^k \delta _i, \Bigl ( \frac{p! \Vert F'(x_0)\Vert _{*} }{(p + 1)L_p2^{p - 1}} \Bigr )^{1 \over p} \biggr \} \end{aligned}$$

### Proof

According to (3.7), one step of RCTM (5.8) provides us with the following guarantee in terms of the subgradients of our objective $\varPhi _{k + 1}(\cdot )$:

5.10 $$\begin{aligned} \begin{array}{rcl} \Vert \varPhi '_{k + 1}(z_t) \Vert _{*}\le & {} \frac{a_{k + 1} (p + 1) L_p}{p!} \Vert \varPhi '_{k + 1}(z_{t - 1}) \Vert _{*}^p, \end{array} \end{aligned}$$

where we used in (3.7) the values $q = 2$, $\sigma _q = 1$, $a_{k + 1} L_p$ for the Lipschitz constant of the *p*th derivative of the smooth part of $\varPhi _{k + 1}$, and $H = a_{k + 1}pL_p$.

Denote $\beta \equiv \left( { a_{k + 1}(p + 1)L_p \over p! } \right) ^{1 \over p - 1} {{\mathop {=}\limits ^{(5.7)}}} \left( { (p + 1) L_p \over 2 \cdot p! \cdot \Vert F'(x_k)\Vert _* } \right) ^{1 \over p}$. Then, from (5.10) we have

5.11 $$\begin{aligned}&\beta \Vert \varPhi '_{k + 1}(z_t) \Vert _{*} \le \bigl (\beta \Vert \varPhi '_{k + 1}(z_{t - 1}) \Vert _{*}\bigr )^{p} \nonumber \\&\quad \le \dots \;\; \le \;\; \bigl (\beta \Vert \varPhi '_{k + 1}(z_0) \Vert _{*}\bigr )^{p^t} \nonumber \\&\quad = (\beta a_{k + 1}\Vert F'(x_k)\Vert _{*})^{p^t} \nonumber \\&\quad = \left( a_{k + 1}^{p \over p - 1} \left( { (p + 1) L_p \over p! }\right) ^{1 \over p - 1} \Vert F'(x_k)\Vert _{*} \right) ^{p^t} \nonumber \\&\quad {{\mathop {=}\limits ^{(5.7)}}} \; \left( {1 \over 2}\right) ^{p^t}. \end{aligned}$$

Therefore, for

5.12 $$\begin{aligned} \begin{array}{rcl} t\ge & {} \log _p \log _2 \left( \frac{1}{\beta \delta _{k + 1}} \right) \; = \; \frac{1}{\log _2 p} \cdot \log _2 \log _2 \left( \frac{1}{ \delta _{k + 1}} \left( { 2 \cdot p! \cdot \Vert F'(x_k) \Vert _* \over (p + 1) L_p } \right) ^{1 \over p} \right) , \end{array} \end{aligned}$$

it holds $\Vert \varPhi '_{k + 1}(z_t)\Vert _{*} \le \delta _{k + 1}$. To finish the proof, let us estimate $\Vert F'(x_k) \Vert _{*}$ from above. We have

5.13 $$\begin{aligned} 2^{3p - 2 \over p} \left( \frac{(p + 1)L_p}{p!} \right) ^{2 \over p} R_k(\delta )&{{\mathop {\ge }\limits ^{(5.3)}}}&2^{2(p - 1) \over p} \left( \frac{(p + 1)L_p}{p!} \right) ^{2 \over p} \sum \limits _{i = 1}^k a_i^2 \Vert F'(x_i)\Vert _{*}^2 \nonumber \\&{{\mathop {=}\limits ^{(5.7)}}}&\sum \limits _{i = 1}^k \Vert F'(x_{i - 1})\Vert _{*}^{2(1 - p) \over p} \Vert F'(x_i)\Vert _{*}^2. \end{aligned}$$

Thus, for every $1 \le i \le k$ it holds

5.14 $$\begin{aligned} \Vert F'(x_i)\Vert _{*} \; {{\mathop {\le }\limits ^{(5.13)}}} \; \Vert F'(x_{i - 1})\Vert _{*}^{\rho } \cdot \mathcal {D}, \end{aligned}$$

with $\mathcal {D} \equiv R_k^{1/2}(\delta ) \left( \frac{(p + 1) L_p}{p!} \right) ^{1 \over p} 2^{3p - 2 \over 2p}$, and $\rho \equiv \frac{p - 1}{p}$. Therefore,

$$\begin{aligned}&\Vert F'(x_k)\Vert _{*} \; {{\mathop {\le }\limits ^{(5.14)}}} \; \Vert F'(x_0)\Vert _{*}^{\rho ^k} \cdot \mathcal {D}^{1 + \rho + \rho ^2 + \dots + \rho ^{k - 1}} \\&\quad = \Vert F'(x_0)\Vert _{*} \cdot \Bigl ( \Vert F'(x_0)\Vert _{*}^{\rho ^k - 1} \cdot \mathcal {D}^{\frac{\; \;1 - \rho ^k}{1 - \rho }} \Bigr ) \\&\quad = \Vert F'(x_0)\Vert _{*} \cdot \left( \frac{\mathcal {D}^{p}}{\Vert F'(x_0)\Vert _{*}} \right) ^{1 - \rho ^k} \; \le \; \Vert F'(x_0) \Vert _{*} \cdot \max \bigl \{ \frac{\mathcal {D}^p}{\Vert F'(x_0)\Vert _{*}}, 1 \bigr \} \\&\quad = \max \biggl \{ \frac{(p + 1) L_p 2^{p - 1}}{p!} \Bigl ( \Vert x_0 - x^{*}\Vert + \sum \limits _{i = 1}^k \delta _i \Bigr )^p, \; \Vert F'(x_0)\Vert _{*} \biggr \}. \end{aligned}$$

Substitution of this bound into (5.12) gives (5.9). $\square$

Let us prove now the rate of convergence for the outer iterations. This is a direct consequence of Theorem 5 and the choice (5.7) of the coefficients $\{ a_{k} \}_{k \ge 1}$.

### Lemma 3

Let for a given $\varepsilon > 0$,

5.15 $$\begin{aligned} \begin{array}{rcl} F(x_k) - F^{*}\ge & {} \varepsilon , \qquad 1 \le k \le K. \end{array} \end{aligned}$$

Then for every $1 \le k \le K$, we have

5.16 $$\begin{aligned} \begin{array}{rcl} F(\bar{x}_k) - F^{*}\le & {} \frac{L_p \left( \Vert x_0 - x^{*} \Vert + \sum _{i = 1}^k \delta _i \right) ^{p + 1} }{k^{p + 1 \over 2}} \frac{(p + 1) 2^{p - 2} V_k(\varepsilon ) }{ p!}, \end{array} \end{aligned}$$

where $$\bar{x}_k \; {\mathop {=}\limits ^{\mathrm {def}}}\; \frac{\sum _{i = 1}^k a_i x_i}{\sum _{i = 1}^k a_i}$$, and $$V_k(\varepsilon ) \; {\mathop {=}\limits ^{\mathrm {def}}}\; \left( \frac{\Vert F'(x_0)\Vert _{*} \cdot ( \Vert x_0 - x^{*}\Vert + \sum _{i = 1}^k \delta _i )}{\varepsilon } \right) ^{p - 1 \over k}$$.

### Proof

Using the inequality between the arithmetic and geometric means, we obtain

5.17 $$\begin{aligned} R_{k}(\delta )&{{\mathop {\ge }\limits ^{(5.3)}}}&\frac{1}{2}\sum \limits _{i = 1}^k a_i^2 \Vert F'(x_i)\Vert _*^2 \; {{\mathop {=}\limits ^{(5.7)}}} \; \frac{1}{8} \left( \frac{p!}{(p + 1)L_p} \right) ^{2 \over p - 1} \sum \limits _{i = 1}^k \frac{a_i^2}{a_{i + 1}^{2p \over p - 1}} \nonumber \\\ge & {} \frac{k}{8} \left( \frac{p!}{(p + 1)L_p} \right) ^{2 \over p - 1} \left( \prod \limits _{i = 1}^k \frac{a_i^2}{a_{i + 1}^{2p \over p - 1}} \right) ^{1 \over k} \nonumber \\= & {} \frac{k}{8} \left( \frac{p!}{(p + 1)L_p} \right) ^{2 \over p - 1} \left( \frac{a_1}{a_{k + 1}} \right) ^{2p \over (p - 1)k} \left( \prod \limits _{i = 1}^k a_i \right) ^{-2 \over (p - 1)k} \nonumber \\\ge & {} \frac{k^{p + 1 \over p - 1}}{8} \left( \frac{p!}{(p + 1)L_p} \right) ^{2 \over p - 1} \left( \frac{a_1}{a_{k + 1}} \right) ^{2p \over (p - 1)k} \left( \sum \limits _{i = 1}^k a_i \right) ^{-2 \over p - 1}. \end{aligned}$$

Therefore,

$$\begin{aligned} F(\bar{x}_k) - F^{*}\le & {} \frac{1}{\sum \limits _{i = 1}^k a_i} \sum \limits _{i = 1}^k a_i (F(x_i) - F^{*}) \; {{\mathop {\le }\limits ^{(5.3)}}} \; \frac{R_k(\delta )}{\sum \limits _{i = 1}^k a_i} \\&{{\mathop {\le }\limits ^{(5.17)}}}&\frac{ R_k(\delta )^{p + 1 \over 2} }{k^{p + 1 \over 2}} \frac{(p + 1) L_p}{p!} \left( \frac{a_{k + 1}}{a_1} \right) ^{p \over k} 8^{p - 1 \over 2} \\= & {} \frac{L_p \left( \Vert x_0 - x^{*} \Vert + \sum _{i = 1}^k \delta _i \right) ^{p + 1} }{k^{p + 1 \over 2}} \frac{(p + 1) 2^{p - 2} }{ p!} \left( \frac{\Vert F'(x_0)\Vert _{*}}{\Vert F'(x_k)\Vert _{*}} \right) ^{p - 1 \over k}, \end{aligned}$$

where the first inequality holds by convexity. At the same time, we have

$$\begin{aligned} \Vert F'(x_k)\Vert _{*}\ge & {} \frac{\langle F'(x_k), x_k - x^{*} \rangle }{\Vert x_k - x^{*}\Vert } \; \ge \; \frac{F(x_k) - F^{*}}{\Vert x_k - x^{*}\Vert } \\&{{\mathop {\ge }\limits ^{(5.15)}}}&\frac{\varepsilon }{\Vert x_k - x^{*}\Vert } \; {{\mathop {\ge }\limits ^{(5.3)}}} \; \frac{\varepsilon }{\Vert x_0 - x^{*}\Vert + \sum _{i = 1}^k \delta _i }. \end{aligned}$$

Thus, $\left( \frac{\Vert F'(x_0)\Vert _{*}}{\Vert F'(x_k)\Vert _{*}} \right) ^{p - 1 \over k} \le V_k(\varepsilon )$ and we obtain (5.16). $\square$

### Remark 1

Note that $\bigl (\frac{1}{\varepsilon }\bigr )^{p - 1 \over k} = \exp \bigl ( {p - 1 \over k} \ln {1 \over \varepsilon } \bigr )$. Therefore after $k = O\left( \ln {1 \over \varepsilon }\right)$ iterations, the factor $V_k(\varepsilon )$ is bounded by an absolute constant.

Since the local convergence of RCTM is very fast (5.9), we can choose the inner accuracies $\{ \delta _i \}_{i \ge 1}$ small enough, to have the right hand side of (5.16) being of the order $\tilde{O}(1 / k^{p + 1 \over 2})$. Let us present a precise statement.

### Theorem 6

Let $\delta _k \equiv \frac{c}{k^s}$ for fixed absolute constants $c > 0$ and $s > 1$. Let for a given $\varepsilon > 0$, we have

$$\begin{aligned} F(x_k) - F^{*}\ge & {} \varepsilon , \qquad 1 \le k \le K. \end{aligned}$$

Then, for every *k* such that $\ln \frac{\Vert F'(x_0)\Vert _{*} R}{ \varepsilon } \le k \le K$, we get

5.18 $$\begin{aligned} \begin{array}{rcl} F(\bar{x}_k) - F^{*}\le & {} \frac{L_p R^{p + 1}}{k^{p + 1 \over 2}} \frac{(p + 1) 2^{p - 2} \exp (p - 1)}{p!}, \end{array} \end{aligned}$$

where

$$\begin{aligned} R \; {\mathop {=}\limits ^{\mathrm {def}}}\; \Vert x_0 - x^{*}\Vert + \frac{cs}{s - 1}. \end{aligned}$$

The total number of oracle calls $N_k$ during the first *k* iterations is bounded as follows:

$$\begin{aligned} N_k\le & {} k \cdot \Bigl ( 1 + \frac{1}{\log _2 p} \log _2 \log _2 \frac{2D k^s }{c} \Bigr ), \end{aligned}$$

where

$$\begin{aligned} D \; {\mathop {=}\limits ^{\mathrm {def}}}\; \max \biggr \{ R, \, \Bigl ( \frac{p! \Vert F'(x_0)\Vert _{*} }{(p + 1)L_p2^{p - 1}} \Bigr )^{1 \over p} \biggl \}. \end{aligned}$$

### Proof

Indeed,

$$\begin{aligned} \sum \limits _{i = 1}^k \delta _i= & {} c\Biggl (1 + \sum \limits _{i = 2}^k \frac{1}{i^s} \Biggr ) \; \; \le \; \; c\Biggl (1 + \int \limits _1^k \frac{dx}{x^{s}} \Biggr ) \; \; = \; \; c\Biggl (1 - \frac{1}{s - 1} \int \limits _1^k dx^{-(s - 1)} \Biggr ) \\= & {} c\Biggl (1 - \frac{k^{-(s - 1)}}{s - 1} + \frac{1}{s - 1} \Biggr ) \; \; \le \; \; \frac{cs}{s - 1}. \end{aligned}$$

Thus, we obtain (5.18) directly from the bound (5.16), and by the fact that

$$\begin{aligned} V_k(\varepsilon )\equiv & {} \Bigl ( \frac{\Vert F'(x_0) \Vert _{*} R}{\varepsilon } \Bigr )^{\frac{p - 1}{k}} \; = \; \exp \Bigl ( \frac{p - 1}{k} \log \frac{\Vert F'(x_0) \Vert _{*} R}{\varepsilon } \Bigr ) \\\le & {} \exp (p - 1), \end{aligned}$$

when $$k \ge \ln \frac{\Vert F'(x_0) \Vert _{*} R }{ \varepsilon } $$.

Finally,

$$\begin{aligned} N_k&{{\mathop {\le }\limits ^{(5.9)}}}&\sum \limits _{i = 1}^k \left\lceil \frac{1}{\log _2 p} \log _2 \log _2 \frac{2 D }{\delta _i} \right\rceil \; \le \; k + \frac{1}{\log _2 p} \sum \limits _{i = 1}^k \log _2 \log _2 \frac{2Di^s}{c} \\\le & {} k + \frac{1}{\log _2 p} \sum \limits _{i = 1}^k \log _2 \log _2 \frac{2Dk^s}{c} \; = \; k \cdot \Biggl (1 + \frac{1}{\log _2 p} \log _2 \log _2 \frac{2Dk^s}{c} \Biggr ). \end{aligned}$$

$$\square $$

Note that we were able to justify the global performance of the scheme, using only the local convergence results for the inner method. It is interesting to compare our approach with the recent results on the path-following second-order methods [5].

We can drop the logarithmic components in the complexity bounds by using the *hybrid proximal methods* (see [15, 16]), where at each iteration only one step of RCTM is performed. The resulting rate of convergence there is $O(1 / k^{p + 1 \over 2})$, without any extra logarithmic factors. However, this rate is worse than the rate $O(1 / k^p)$ provided by the Theorem 3 for the primal iterations of RCTM (3.1).
